# Subtropical marine reserve as key habitats for the critically endangered sand tiger shark (*Carcharias taurus*) in the Southwestern Atlantic

**DOI:** 10.1111/jfb.70479

**Published:** 2026-04-27

**Authors:** Ana Clara S. Athayde, Guilherme Bertuzo, Otto Bismarck F. Gadig, Rafael R. Munhoz, Letícia M. Fukushima, Caio R. Pimentel, Fernanda A. Rolim, Guilherme H. Pereira‐Filho, Fabio S. Motta

**Affiliations:** ^1^ Laboratório de Ecologia e Conservação Marinha (LABECMar), Instituto do Mar, Universidade Federal de São Paulo (UNIFESP), Rua Dr. Carvalho de Mendonça Santos São Paulo Brazil; ^2^ Programa de Pós‐Graduação em Aquicultura e Pesca do Instituto de Pesca SAA, Avenida Conselheiro Rodrigues Alves São Paulo São Paulo Brazil; ^3^ Laboratório de Pesquisa em Elasmobrânquios (ELASMOBRASIL), Instituto de Biociências, Universidade Estadual Paulista (UNESP), Campus do Litoral Paulista, Praça Infante Dom Henrique, s/n ‐ Parque Bitaru São Vicente São Paulo Brazil

**Keywords:** Brazilian coast, elasmobranchii, no‐take zone, population, reproductive biology, stéreo‐video, SWA

## Abstract

This study documents the occurrence, seasonal patterns and reproductive activity of the sand tiger shark (*Carcharias taurus*) within a no‐take marine protected area in southeastern Brazil, using Baited Remote Underwater Video (BRUV) and diving records. We recorded the species in the Alcatrazes Archipelago during both summer and winter, including pregnant females and reproductively active, non‐pregnant females with fresh mating wounds. These findings enhance understanding of the population dynamics of this critically endangered shark and highlight the ecological and conservation value of marine protected areas in supporting individuals across life stages and reproductive conditions year‐round.

The sand tiger shark, *Carcharias taurus* Rafinesque, 1810 (Lamniformes: Odontaspididae), is a large (up to 3.2 m total length, TL) coastal migratory shark species distributed throughout temperate and subtropical waters worldwide (except in the Eastern Pacific). In the Southwestern Atlantic, this species range extends from 20.8° S to 41° S (Cuevas et al., 2021; Ebert et al., 2021). The prevailing literature indicates that, after mating in the cooler waters of Argentina and Uruguay, pregnant females aggregate in subtropical waters off Brazil (Lucifora et al., 2002; Sadowsky, 1967), whereas newborns and young juveniles inhabit shallow coastal zones in southern Brazil, Uruguay and Argentina (Cardoso et al., 2010; Cuevas et al., 2021; Mayer et al., 2025; Silveira et al., 2018; Vooren & Klippel, 2005).

Seasonal fishing patterns have also provided insights into the species' movements, as *C. taurus* was historically targeted in Brazil during the so‐called ‘Mangona’ season, when individuals move closer to the coast (Hornke, 2017; Santos et al., 2019). However, this seasonal targeting, coupled with incidental captures across its distribution, has made populations highly susceptible to overfishing. The species has been captured both intentionally and unintentionally by artisanal, recreational and industrial fisheries, resulting in severe population declines (García et al., 2008; Lucifora, 2003). Globally, *C. taurus* is listed as Critically Endangered on the IUCN Red List (Rigby et al., 2021), and in Brazil, fisheries data indicate a population decline of at least 70% between 1970 and 2010 (Barreto, 2021; Montealegre‐Quijano, 2021). Consequently, the species is also classified as Critically Endangered by the Brazilian government (MMA, 2022; MMA, 2018), and its capture, retention and trade have been prohibited since 2014 (Brasil, 2014; Kotas et al., 2018).

Given the critical conservation status, understanding this species' occurrence within a marine protected area (MPA) network, and how these areas are used by them, including diel and seasonal cycles, provides valuable insight for evaluating the network's effectiveness and guiding adaptive management (Hooker et al., 2011; MacKeracher et al., 2019). Here, we used a 4‐year dataset collected in one of the largest no‐take MPAs over the Southwestern Atlantic continental shelf to document the occurrence, seasonality and reproductive activity of *C. taurus* in the region, contributing to our understanding of the ecology of this endangered shark and providing key information for its conservation and management.

The sampling site is located off the northern coast of São Paulo state, southeast Brazil, approximately 33 km from the mainland (24° 10′ S, 45° 70′ W; Figure 1), and encompasses two no‐take MPAs: the Tupinambás Ecological Station (TES, IUCN Category Ia), created in 1987 (24.63 km^2^), and the Alcatrazes Archipelago Wildlife Refuge (AWR, IUCN Category III), established in 2016 (674.09 km^2^). These MPAs comprise islands, islets and submerged rocky reefs in the Southwestern Atlantic, which sustain high biodiversity, including several endemic terrestrial and marine species (Lanna et al., 2007; Nogueira et al., 2001). The archipelago is characterized by the predominance of sandy sediments (Hoff et al., 2015) and rocky reefs covered by macroalgae, turf filamentous algae, encrusting coralline algae, sponges and corals (Aued et al., 2018).

**FIGURE 1:**
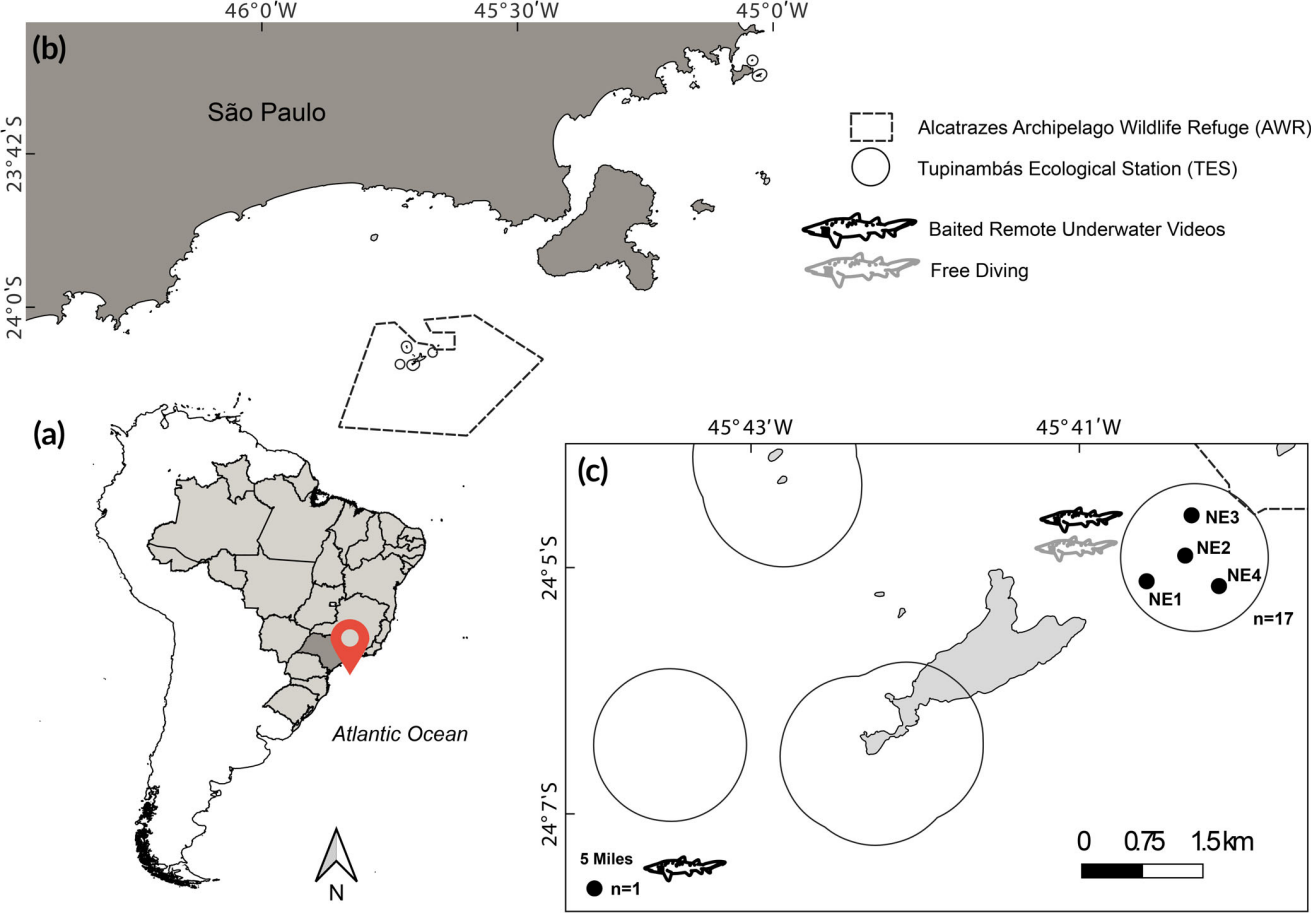
Study area showing the Alcatrazes Archipelago Wildlife Refuge (AWR) and the Tupinambás Ecological Station (TES), located off the coast of São Paulo state, southeastern Brazil. (a) Location of the study area along the Brazilian coast. (b) Overview of AWR and TES in São Paulo state. (c) Detailed view of sampling sites. Sampling locations are indicated by points; black shark silhouettes represent Baited Remote Underwater Video (BRUV) deployments, and grey silhouettes represent free diving surveys.

Baited Remote Underwater Stereo‐Video Systems (Stereo‐BRUVs; see details in Motta et al., 2024) were used to assess the relative abundance of *C. taurus* within the AWR. A total of 315 deployments were conducted at 38 sites, ranging from 2 to 50 m in depth across the archipelago, and surveyed during austral summer and winter between 2022 and 2025 as part of the long‐term biodiversity monitoring program, Mar de Alcatrazes. Sharks were counted and measured (total length), following all length rules recommended for configuring stereo measurements detailed in Langlois et al. (2020). Additionally, a citizen science approach was incorporated to gather complementary data on *C. taurus* in the area, since the AWR is visited by recreational free and scuba divers. During summer 2024, we recorded *C. taurus* through free diving with GoPro Hero 10 Black® cameras at depths between 5 and 10 m. For individual identification and documentation, a photographic identification methodology was applied, enabling the recognition of sex and maturity (when possible), and distinctive body marks. The species was identified despite limited visibility, as *C. taurus* is easily distinguished from other large coastal sharks in the Southwestern Atlantic by the size and relative position of its fins (see Ebert et al., 2021). Pregnancy was inferred based on the presence of coelomic distension (Bansemer & Bennett, 2009), and mating bite marks on females were described following Wyffels et al. (2025).

Nine *C. taurus* individuals were recorded by BRUVs in the AWR (Figure 2; Table S1 and Video S1). The first record, a single 1.89 m TL male, was obtained in July 2022, observed at 36 m depth at a submerged rock reef (Northeast Reef; NE1) site, located on the northeastern edge of the archipelago. Given the extensive size of this reef, four sampling sites were established in the area (NE1, NE2, NE3 and NE4). In July 2024, four individuals were observed at three sites: one at Northeast Reef 1 (NE1, 30 m deep; unsexed), two at Northeast Reef 2 (NE2, 40 m deep; 1.87 m TL male and 2.1 m TL suspected pregnant female as noted by the visible lateral abdominal bulge; Figure 2a) and one at the submerged rock reef 5 Miles (45 m deep; unsexed). In July 2025 (winter), again at NE1, four individuals were recorded at 20 m depth. This group consisted of three males (1.78, 1.88 and 1.93 m TL) and one female (2.11 m TL). In December 2024, citizen science data from recreational diving reported a group of nine *C. taurus* individuals in the Northeast Reef, at 5–8 m depth (Figure 2; Table S1 and Video S2). The group included two larger adult females (~2.5 and 2 m TL) and two smaller females (~1.5 m TL), one male (~1.5 m TL) and four unsexed individuals (~1.5 m TL). In addition, one of the females (2 m TL) exhibited bite marks on the body (Figure 2j). These marks (several bright white lacerations forming an arc pattern) are concentrated in the anterior region of the body, dorsally (pre first dorsal fin, corresponding to upper teeth) and laterally (over the right pectoral fin base, corresponding to lower teeth). Regarding maturity stage, most specimens were classified as subadults or adults based on Sadowsky (1967) (Table S1).

**FIGURE 2:**
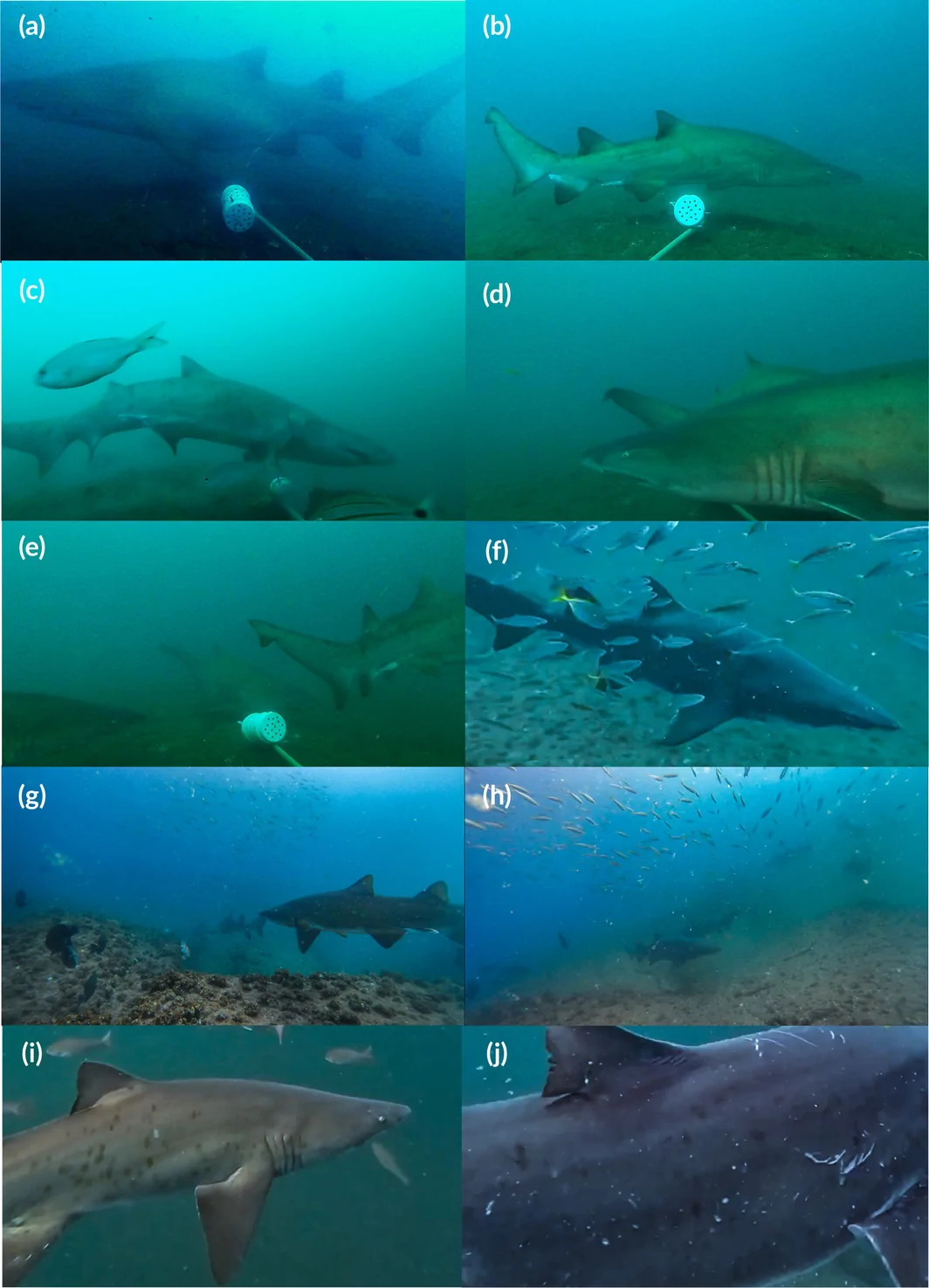
*Carcharias taurus* individuals recorded by Baited Remote Underwater Video (BRUVs) (a–e); and free diving (f–j), including a pregnant female (a) and a female with visible fresh mating wounds, evidenced by white semicircular lacerations and puncture wounds (j).

Our findings provide new insights into the reproductive ecology of *C. taurus* in the Southwestern Atlantic, particularly within the AWR. Records obtained during both austral summer (December) and winter (July) are consistent with the reproductive cycle previously described for the species in the Southwestern Atlantic (Lucifora et al., 2002). In this region, pregnant females are mainly reported in southern Brazil from January to June (Sadowsky, 1967), whereas reproductively active but non‐pregnant females occur in Argentine waters during January and April (Lucifora et al., 2002). At AWR, subadult and adult males and females were recorded during summer, including one female with fresh mating wounds, providing evidence of reproductive activity in the area. The characteristics of these mating lesions match level‐3 injuries (where the wound bed is bright white, lacks pigment and the underlying tissue is not visible) described by Wyffels et al. (2025) for a western North Atlantic population, indicating that mating likely occurred 29–40 days prior to observation. During winter, an adult female exhibiting a distended coelom and lateral bulges suggestive of pregnancy was also recorded, suggesting the presence of females at different reproductive stages within the AWR. Considering the similarities in mating‐wound healing patterns among populations, the occurrence of a recently mated female in summer and a pregnant female in winter is consistent with Sadowsky (1967), who indicated that mating and the onset of gestation occur along the southern coast of São Paulo (southeastern Brazil) during late spring and early summer. According to this author, early‐stage embryos (<9 cm TL) are found in January, whereas females carrying larger embryos (60–69 cm TL) occur during the austral winter. Sadowsky (1967) further suggested that parturition likely takes place off the southern São Paulo coast, as the maximum embryo sizes recorded in coastal areas (up to 70 cm TL, rarely 80 cm) are smaller than the species' size at birth (90–100 cm TL; Kotas et al., 2018; Ebert et al., 2021), indicating that birth likely occurs elsewhere. Although nursery areas are known to occur along the southeastern Brazilian coast (Lucifora et al., 2002; Sadowsky, 1967), their precise boundaries remain unknown. Nevertheless, these patterns highlight the importance of MPAs in safeguarding coastal sharks, particularly species that depend on specific habitats for reproduction or feeding (Speed et al., 2010). According to Lucifora et al. (2002), during winter, males may migrate offshore to continental shelf waters since they are not observed in large numbers in coastal waters of Argentina, Uruguay and southern Brazil. In this context, our findings help elucidate patterns of offshore migration in Brazil, suggesting that males may be directed toward reef and insular environments, presumably characterized by high food availability, such as AWR (see Motta et al., 2021, 2024).

The ecological features of AWR are consistent with habitat characteristics highlighted as important for the species elsewhere. Shallow inshore habitats, particularly those with rocky formations such as caves, reefs or islands, are known to be suitable for *C. taurus* aggregations, being used for feeding, mating and parturition (Bansemer & Bennett, 2009; Mayer et al., 2025; Price et al., 2024). Most of our observations occurred in shallow waters, especially in summer, in line with the bathymetric preference reported for nurseries and feeding grounds in other regions (Dicken et al., 2006; Kneebone et al., 2014; Mayer et al., 2025). Temperature may also play a crucial role (Mayer et al., 2025), as it influences habitat suitability and reproductive behaviour across latitudes (Simpfendorfer & Heupel, 2012; Sunday et al., 2012). In the Southwestern Atlantic, pregnant females are thought to migrate northwards toward warmer waters in Brazil for gestation between April and May (Lucifora et al., 2002). Our winter record of a pregnant female in AWR supports this hypothesis, suggesting that the archipelago may provide favourable thermal conditions for early gestation.

Similar to other shark species, *C. taurus* demonstrate philopatry to certain areas (Hueter et al., 2005). Despite their ability to travel long distances, many migratory shark species exhibit strong site fidelity to specific geographical areas, such as feeding grounds (Anderson et al., 2011; Barnett et al., 2011; Reynolds et al., 2017), breeding sites and nursery habitats (Dwyer et al., 2019; Feldheim et al., 2014; Ogburn et al., 2018). Although we did not record neonates or juveniles, the presence of reproductively active individuals and a suspected pregnant female aligns with patterns reported for Uruguay, where sequential occurrences of adults (including gravid females) followed by juveniles suggest that rocky and shallow habitats act as ‘stepping stones’ for reproductive aggregation and connectivity (Laporta et al., 2018; Mayer et al., 2025; Silveira et al., 2018). These observations indicate that for migratory sharks like *C. taurus*, AWR may represent an ecologically important site within the broader network of subtropical coastal habitats that support different stages of the *C. taurus* life cycle.

Coastal environments are crucial for the survival of many threatened shark and ray species, as a significant portion of their populations inhabit these areas. However, these same habitats are among the most vulnerable to human‐driven pressures, including overfishing, habitat degradation, climate change and pollution (Dulvy et al., 2021). In southern Brazil, Uruguay and Argentina, fisheries targeting coastal sharks exhibit marked seasonal peaks during the warmer months, particularly from November to February (Cuevas et al., 2021; Hornke, 2017; Irigoyen & Trobbiani, 2016; Santos et al., 2019; Vooren & Klippel, 2005). As a result of intense exploitation, populations of *C. taurus* in the Southwestern Atlantic are estimated to have declined by more than 80% over the past seven decades, mirroring trends observed in other parts of the world (Rigby et al., 2021). Given that many shark species are large‐bodied, slow‐growing and reproduce late in life, ensuring effective protection of mature and pregnant females is critical for population recovery (Chin et al., 2023; Otway et al., 2004).

The establishment of MPAs has become a widely adopted ecosystem‐based management strategy to prevent further declines in shark populations (Chin et al., 2023; MacKeracher et al., 2019; Rigby et al., 2019). These areas operate by restricting major threats to species and habitats within defined boundaries, thereby reducing mortality and supporting population recovery (Dwyer et al., 2020). Although resource managers face challenges due to the high mobility of migratory marine species (Dwyer et al., 2019), networks of smaller MPAs positioned along migration corridors at locations where individuals predictably aggregate can still provide conservation benefits by protecting critical habitats and reducing exposure to threats (Lynch et al., 2013; MacKeracher et al., 2019; Rigby et al., 2019). In this context, the Laje de Santos Marine State Park (Laje MSP ‐ IUCN Category II) and Queimada Grande Island (QGI – IUCN Category V) also serve as important areas for endangered shark species (Athayde et al., 2026; Luiz et al., 2008). Sighting records of *C. taurus* in Laje MSP (July 2010, December 2016, September 2017 and December 2024; unpublished data; Video S3), in QGI (dating back to the summer of 1998) and in the AWR during both summer and winter periods suggest that these areas may be ecologically connected, potentially acting as key sites within a broader migratory corridor for this endangered species along the southeastern Brazilian coast.

Recent studies have highlighted the ecological and conservation importance of the AWR, particularly in protecting endangered shark species and providing suitable habitats for their occurrence and aggregation (Corrêa et al., 2025; Meira et al., 2024; Motta et al., 2024). The MPA has recently been designated as an Important Shark and Ray Area (ISRA; Jabado et al., 2025), a framework developed to identify and map critical habitats for key life‐history processes of chondrichthyans worldwide (Hyde et al., 2022). Our findings provide additional support for this designation, reinforcing criterion A (vulnerability), sub‐criteria C1 (reproductive areas) and C5 (undefined aggregations), while also contributing to criterion C4 (movement), which encompasses areas regularly or predictably used during migrations that promote connectivity among functionally important habitats. Assessing how sharks occupy sites within an MPA network, and how these patterns compare across interspecific, circadian, temporal and ontogenetic differences, provides a basis for evaluating the network's effectiveness and informs adaptive management approaches (Dwyer et al., 2023; Hooker et al., 2011; MacKeracher et al., 2019).

Our findings highlight the ecological and conservation importance of the AWR for *C. taurus*, demonstrating that the area supports individuals at different life stages and reproductive conditions throughout the year. By providing suitable habitat for mating, early gestation and aggregation, AWR likely functions as a critical node within the broader subtropical coastal network of habitats. These results reinforce the value of no‐take MPAs in protecting coastal sharks and contribute to understanding the spatial and temporal dynamics of this endangered species, supporting effective management and conservation strategies along the southeastern Brazilian coast.

## AUTHOR CONTRIBUTIONS

**Ana Clara S. Athayde:** Conceptualization; data collection; formal analysis; writing—original draft; writing—review and edition. **Guilherme Bertuzo, Otto Bismarck F. Gadig, Rafael R. Munhoz, Letícia M. Fukushima, Caio R. Pimentel and Fernanda A. Rolim:** Conceptualization; data collection and writing—review and editing. **Guilherme H. Pereira‐Filho:** Writing—review and editing. **Fabio S. Motta:** Conceptualization; data collection; writing—review and editing; supervision; project administration; funding acquisition. All authors read and approved the final version of the manuscript.

## FUNDING INFORMATION

Financial support was provided by Petrobras (Tripartite Agreement FapUnifesp‐Unifesp—Petrobras #23089102938/2019‐54—Mar de Alcatrazes Project) and FAPESP (Process Number 2023/11845‐3). OBF Gadig, GH Pereira‐Filho and FS Motta acknowledge individual grants from the Brazilian Research Council (CNPq).

## Supporting information


**Table S1:** In this table, all sharks' records are presented. The table presents the following table header ‘Individual records of the species across sampling years (2022–2025) in the Alcatrazes Archipelago, obtained through BRUVs and underwater surveys. For each sighting, month/year, method, site, total length, sex, maturity stage and depth are provided’.

**Video S1.** In this video, all BRUV records of the sand tiger shark used in this study are presented.

**Video S2.** In this video, the free diving record of the sand tiger shark used in this study is presented.

**Video S3.** In this video, we have compiled three recordings of sand tiger sharks made in the Laje de Santos Marine State Park (Laje MSP). All videos are available on the open social network You Tube.

## Data Availability

The data that support the findings of this study are available from the corresponding author upon reasonable request.
