# Supplementary material for: Subtropical marine reserve as key habitats for the critically endangered sand tiger shark (Carcharias taurus) in the Southwestern Atlantic
Source: J Fish Biol. 2026 Apr 27;109(2):1290–6. doi: 10.1111/jfb.70479 (PMC13521696; doi:10.1111/jfb.70479)
Supplement: Supplementary file 1 [file JFB-109-1290-s004.docx]

Table S1. Individual records of the species across sampling years (2022–2025) in the Alcatrazes Archipelago, obtained through BRUVs and underwater surveys. For each sighting, month/year, method, site, total length, sex, maturity stage, and depth are provided.

| **Month/Year** | **Record Method** | **Site** | **Length** | **Sex** | **Maturity stage** | **Depth** |
| --- | --- | --- | --- | --- | --- | --- |
| July/2022 | BRUV | NE1 | 1.89m | Male | Adult | 36m |
| July/2024 | BRUV | 5 milhas 1 | - | Unsexed | - | 45m |
| July/2024 | BRUV | NE2 | 2.1m | Female | Adult (pregnant) | 40m |
| July/2024 | BRUV | NE2 | 1.87m | Male | Adult | 40m |
| July/2024 | BRUV | NE1 | - | Unsexed | - | 30m |
| December/2024 | Diving | NE | 2.5m | Female | Adult | 5-8m |
| December/2024 | Diving | NE | 2 m | Female | Adult (mating scars) | 5-8m |
| December/2024 | Diving | NE | 1.5m | Female | Subadult | 5-8m |
| December/2024 | Diving | NE | 1.5m | Female | Subadult | 5-8m |
| December/2024 | Diving | NE | 1.5m | Male | Subadult | 5-8m |
| December/2024 | Diving | NE | 1.5m | Unsexed | Subadult | 5-8m |
| December/2024 | Diving | NE | 1.5m | Unsexed | Subadult | 5-8m |
| December/2024 | Diving | NE | 1.5m | Unsexed | Subadult | 5-8m |
| December/2024 | Diving | NE | 1.5m | Unsexed | Subadult | 5-8m |
| July/2025 | BRUV | NE1 | 2.11m | Female | Adult | 20m |
| July/2025 | BRUV | NE1 | 1.93m | Male | Adult | 20m |
| July/2025 | BRUV | NE1 | 1.78m | Male | Subadult | 20m |
| July/2025 | BRUV | NE1 | 1.88m | Male | Adult | 20m |
